# Bulk and single-cell RNA-sequencing analyses revealed potential key genes and the role of CCL19/CCL21-CCR7 axis in hidradenitis suppurativa

**DOI:** 10.1371/journal.pone.0322565

**Published:** 2025-06-02

**Authors:** Xiaodong Lai, Yan Yang, Haini Zhang, Chong Zhang, Meng Wang, Wanxin Chen, Baoxi Wang, Yan Yan

**Affiliations:** Department of Dermatology, Plastic Surgery Hospital, Chinese Academy of Medical Sciences and Peking Union Medical College, Beijing, China; University of Illinois, UNITED STATES OF AMERICA

## Abstract

Hidradenitis suppurativa (HS) is a chronic inflammatory skin disorder, affecting the pilosebaceous unit in apocrine gland-rich areas, characterized by painful nodules, abscesses and draining tunnels. The underlying molecular and immunological mechanisms remain poorly understood. This study aimed to identify key gene expression patterns, hub genes, and analyze the potential role of the CCL19/CCL21-CCR7 axis in HS lesions and peripheral blood using bulk and single-cell RNA sequencing analyses. By employing an integrative approach that included three machine learning methods and subsequent validation on an independent dataset, we successfully identified *AKR1B10, IGFL2, WNK2, SLAMF7,* and *CCR7* as potential hub genes and therapeutic targets for HS treatment. Furthermore, our study found that CCL19 and CCL21 may originate from various cells such as fibroblasts and dendritic cells, playing a crucial role in recruiting CCR7-associated immune cells, particularly Treg cells. The involvement of the CCL19/CCL21-CCR7 axis in HS pathogenesis suggests that other CCR7-expressing cells may also be recruited, contributing to disease progression. These findings significantly advance our understanding of HS pathogenesis offer promising avenues for future CCR7-targeted therapeutic interventions.

## Introduction

Hidradenitis Suppurativa (HS) is a chronic inflammatory skin disease characterized by nodules, abscesses and tunnels [1]. It affects approximately 0.4% of the Western population [2]. The etiology of HS is multifactorial, involving immune dysfunction, genetic predisposition, and alterations in the microbiome, although the precise mechanisms remain incompletely understood [3,4]. HS considerably diminishes quality of life, leading to physical and psychological challenges including severe pain, the inability to work, sexual dysfunction, social withdrawal, and depression [5].

Treating HS is challenging due to its recurring nature and our limited understanding of its pathogenesis [6,7]. Therefore, it is crucial to identify new candidate genes and potential therapeutic targets to improve the prognosis of patients with HS. Previous transcriptomic studies have analyzed a substantial amount of data related to HS [8–11], but these studies are still somewhat limited in terms of sample size and scope. Our study further integrates multiple datasets and employs machine learning algorithms, providing a more robust approach to identify key pathogenic genes.

In this study, we identified differentially expressed genes (DEGs) and performed DEGs enrichment analysis between HS and healthy samples. Three machine learning algorithms were screened for potential biomarkers associated with HS. In addition, we explored the relationship between immune cell infiltration and potential biomarkers in HS. This characterization was further validated using an additional validation cohort and single-cell transcriptome data. In conclusion, our investigation revealed that *AKR1B10, IGFL2, WNK2, SLAMF7,* and *CCR7* likely play a role in various pathological processes associated with HS. Furthermore, we propose that the CCL19/CCL21-CCR7 axis is crucial to the pathogenesis and progression of HS.

## Materials and methods

### Bulk RNA-seq data analysis

The bulk RNA sequencing data of hidradenitis suppurativa and healthy control samples were downloaded from four Gene Expression Omnibus (GEO) datasets, with additional details provided in Table 1. Gene expression values were normalized using the Transcripts per Million (TPM) method. Differentially expressed genes (DEGs) between the HS and healthy control groups were identified using three methods: limma, DESeq2, and edgeR, based on read count values. The screening criteria were set as |log2FC| ≥ 1 and adjusted p-value<0.05.

**Table 1:** Details of GEO datasets used in the study.

GEO Series	Type	Sample	Detailed information	R
GSE154773	RNA-seq	blood skin	blood：21 HS patients and 10 healthy controls skin：22 HS lesions and 10 healthy controls	[44]
GSE155176	RNA-seq	skin	26 HS lesions and 16 healthy controls	[7]
GSE213761	RNA-seq	skin	16 HS lesions and clinical data	UP
GSE128637	Microarray	skin	10 HS lesions and 11 healthy controls (validation cohort)	[45]
GSE220116	ScRNA-seq	skin	5 HS lesions and 10 healthy controls	[46]
GSE175990	ScRNA-seq	skin	3 HS lesions and 1 healthy controls	[30]
GSE154775	ScRNA-seq	skin	5 HS lesions	[44]

GEO: Gene Expression Omnibus; HS: hidradenitis suppurativa; R: reference; UP: unpublished.

### ScRNA-seq data analysis

The single-cell RNA sequencing data of hidradenitis suppurativa and healthy control skin tissue from three publicly available datasets (GSE220116, GSE175990, and GSE154775) from the GEO database were incorporated by Seurat package [12]. Cells with less than 200 genes, mitochondrial counts greater than 25%, and genes expressed in less than three cells were filtered out for quality control. The data was first normalized by functions NormalizeData and ScaleData functions. Then, the FindVariable function was applied to select the top 3000 variable genes. We conducted principal component analysis (PCA) using the top 3000 variable genes. We selected the top 30 principal components (PCs) for sub-clustering and set the resolution parameter to 0.1. To mitigate batch effects and nonbiological technical biases, we employed the Harmony package in Seurat [13]. Default parameter values were utilized for Harmony settings. The expression of known marker genes was used as a reference for annotation of different cell types. The results were visualized by Uniform Manifold Approximation and Projection (UMAP).

### Functional enrichment analysis

Gene ontology (GO) annotation and Kyoto Encyclopedia of Genes and Genomes (KEGG) pathway enrichment analysis were performed using the R package clusterProfiler [14]. An adjusted p-value < 0.05 was set as the threshold for the identification of related GO functions and KEGG pathways.

### Machine learning algorithms to screen hub gene

To identify key biomarkers, we conducted further screening using three widely recognized machine learning algorithms: LASSO (Least Absolute Shrinkage and Selection Operator), SVM-RFE (Support Vector Machine- Recursive Feature Elimination), and RF (Random Forest). To enhance stability and reliability, all machine learning algorithms were subjected to tenfold cross-validation.

We utilized the glmnet R package with tenfold cross-validation [15]. The cv. glmnet function was employed to majorize lambda. The model was specifically tailored for binomial classification using the “binomial” and “class” settings. Based on the minimum lambda value, glmnet was applied to implement LASSO with alpha and a “binomial” method on the training sets. This procedure allowed us to identify and select the most relevant features through the LASSO algorithm for further analysis.

Random Forest was utilized to classify the significant genes using the “randomForest” R package [16]. The random forest analysis, employing a decision tree algorithm, determined the most important variables. Following the construction of a random forest model with 5000 trees on the train cohorts, we identified the optimal number of trees through cross-validation errors. Genes were then ranked by importance, and the 15 most significant genes were selected.

SVM-RFE was employed for recursive feature elimination. Utilizing the “e1071” and “MSVM-RFE” packages for SVM modeling, SVM-RFE implemented sequential backward feature elimination to identify the optimal hub gene [17]. All shared genes were incorporated into our SVM model using 10-fold cross-validation for the training dataset.

Ultimately, we selected the intersection of the three machine learning algorithms as the hub genes of HS. Boxplots of gene expression were generated to compare differences between HS and normal groups. Receiver Operating Characteristic (ROC) curves were constructed to assess the diagnostic value of these candidate genes. The Area Under the Curve (AUC) was calculated, with an AUC value exceeding 0.8 considered an ideal diagnostic threshold.

### Immune microenvironment analysis

The CIBERSORT method is a widely employed technique for assessing immune cell types within the tissue microenvironment [18]. Based on support vector regression, this method deconvolutes the expression matrix of immune cell subtypes, identifying 22 distinct cell types. In this study, the CIBERSORT algorithm was applied to analyze data from HS patients, aiming to infer the relative proportions of the 22 distinct immune-infiltrating cell types. Subsequently, Spearman correlation analysis was conducted to examine the relationship between gene expression and the content of immune cells.

### Specimen collection

All samples were collected between 31 March 2020 and 30 June 2024. All subjects have given written informed consent to the publication of their case details. This study was approved by the Ethics Committee of the Plastic Surgery Hospital of the Chinese Academy of Medical Sciences [2020-16]. All experiments were performed in accordance with relevant guidelines and regulations. HS skin samples were obtained from the center of active inflammatory lesions derived from surgically discarded tissues, defined as lesional skin based on previously published criteria [19]. Healthy control skin, matched for criteria such as age, sex, and ethnicity, was obtained from healthy donors undergoing abdominoplasty. All patients provided written informed consent to the publication of their case details. The clinical characteristics of participants are provided in S1 Table.

### Real-time PCR assay

Total RNA was extracted from HS skin lesions using TRIzol™ Reagent (Invitrogen). Subsequently, the RNA underwent reverse transcription into cDNA employing RevertAid First Strand cDNA Synthesis Kit (Thermo Scientific). Real-time PCR(RT-PCR) assays were performed on the LightCycler® 96 Instrument utilizing Taq Pro Universal SYBR qPCR Master Mix (Vazyme). Result analysis was conducted using the 2^-△△^^CT^ method. The primer sequences employed are as follows: *GAPDH*, forward 5’- agggctgcttttaactctggt -3’ and reverse 5’- ccccacttgattttggaggga -3’; *AKR1B10*, forward 5’- gtgacaccagcacgcattg -3’ and reverse 5’- gcattgaagggatagtcttccaa -3’; *TMPRSS6*, forward 5’- gcagtctgcgtgtactcaatc -3’ and reverse 5’- gctggagttgtagtaagttccc -3’; *SLAMF7*, forward 5’- acaacccctcttgtcaccata -3’ and reverse 5 ‘- cccacatagtagatccctgagtc -3’; *PLEKHG7*, forward 5 ‘- actcgatcagcctattccact -3’ and reverse 5’- aagaatgctgctatacactgtcg -3’; *IGFL2*, forward 5’- agctgaaggttcagggtgtg -3’ and reverse 5’- gggaaaacgtcttctgctttca -3’; *WNK2*, forward 5’- cgcttcctcaagttcgacatc -3’ and reverse 5’- tggactcccagaagtcgtaga -3’.

### Collection of blood samples

Peripheral blood samples were obtained from patients and healthy volunteers by venipuncture into EDTA-coated tubes to prevent coagulation. The blood was processed using a SepMate™-50 kit (STEMCELL Technologies, 85460). The samples were diluted with an equal volume of PBS and gently mixed. The tubes were then centrifuged at 1200 × g for 10 minutes at room temperature. After centrifugation, three distinct layers were observed in the tube: plasma at the top, peripheral blood mononuclear cells (PBMCs) at the interface, and erythrocytes at the bottom. The PBMCs were carefully extracted and transferred to a new centrifuge tube for further processing. The plasma was collected and stored at -80°C until further analysis.

### Flow cytometry analysis

PBMCs were isolated using a Ficoll-Paque gradient centrifugation method. Cells were stained with fluorescently labeled monoclonal antibodies targeting surface markers of interest, including CD45(BioLegend,368526), CD3 (BioLegend,300460), CD4 (BioLegend,357424), CD8 (BioLegend,301012), CD25 (BioLegend,356104), CD127 (BioLegend,351322) and CCR7 (Thermo Fisher Scientific, 17-1979-42). Multicolor flow cytometry was performed using a BD FACS Canto plus, and data were analyzed with NovoExpress (1.6.2) for cell population gating and quantification of marker expression.

### Enzyme-linked immunosorbent assay

Plasma levels of CCL19 (BOSTER, EK0456) and CCL21 (BOSTER, EK0555) were quantified using a commercially available ELISA kits according to the manufacturer’s protocol. Briefly, 96-well plates were coated with capture antibodies specific for the target proteins, followed by incubation with patient plasma samples and detection antibodies conjugated to horseradish peroxidase (HRP). Absorbance was measured at 450 nm using a microplate reader (Thermo Fisher Scientific, Multiskan FC), and concentrations were calculated against a standard curve.

### Statistical analysis

All data processing, statistical analysis, and plotting were performed in R software (version 4.2.0) and Graphpad prism (version 9.0.0). Correlations between two continuous variables were assessed by Spearman correlation coefficients. Differential analysis was realized via the Wilcox test. All statistical analyses were two sided with P < 0.05 considered statistically significant.

## Results

### Analysis and screening of differential expression genes (DEGs) and Biological and functional enrichment analysis

**Fig 1:**
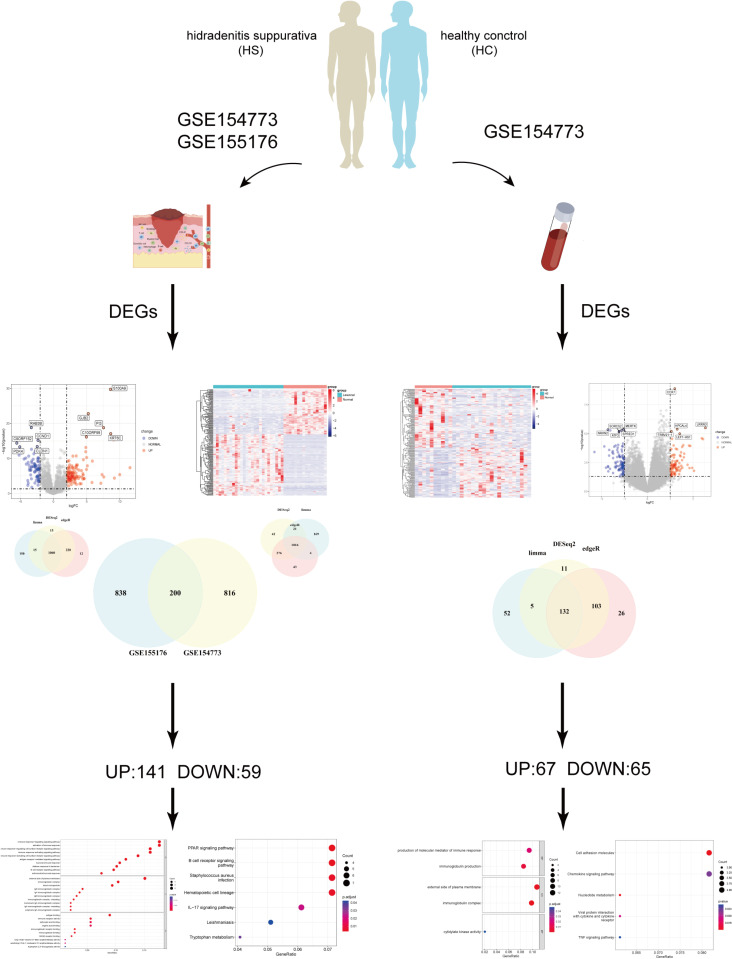
Differentially expressed genes between between hidradenitis suppurativa and healthy samples in both blood and lesion tissues.

To identify DEGs between HS and healthy controls, two bulk RNA sequencing data, GSE154773 and GSE155176, were collected. Three methods, limma, DESeq2, and edgeR were used along with Benjamini-Hochberg (BH) method correction for multiple comparisons. The results from the three methods were intersected to identify reliable DEGs associated with HS. The analysis results of the GSE154773 dataset exhibited that 574 and 442 genes are significantly upregulated and downregulated in the HS lesion, respectively. In the GSE155176 dataset, 682 genes were upregulated, and 378 genes were downregulated in HS lesions. Then we intersected these DEGs in two datasets and identified 200 common DEGs there are 141 upregulated genes and 59 downregulated genes (Fig 1). To gain further insight into the biological functions of these DEGs, we performed KEGG pathway analysis and GO functional analysis. GO analysis revealed that these DEGs are significantly associated with multiple biological processes, and we show the top ten results (Fig 1). The KEGG pathway analysis revealed enrichment in the PPAR signaling pathway, B cell receptor signaling pathway, Staphylococcus aureus infection, hematopoietic cell lineage, IL-17 signaling, tryptophan metabolism, and leishmaniasis (Fig 1). Additionally, 132 DEGs were screened in the HS peripheral blood, including 67 upregulated genes and 65 downregulated genes (Fig 1). Similarly, KEGG pathway analysis and GO functional analysis were employed in peripheral blood sample. GO results indicate that genes related to HS peripheral blood may be involved in biological functions such as the production of molecular mediators of immune response, immunoglobulin production, external side of plasma membrane, immunoglobulin complex, and cytidylate kinase activity (Fig 1). The KEGG pathway analysis showed that Cell adhesion molecules, Chemokine signaling pathway, Nucleotide metabolism, Viral protein interaction with cytokine and cytokine receptor and TNF signaling pathway were enriched by these DEGs (Fig 1).

### Identify hub genes as the characteristic DEGs of HS

**Fig 2:**
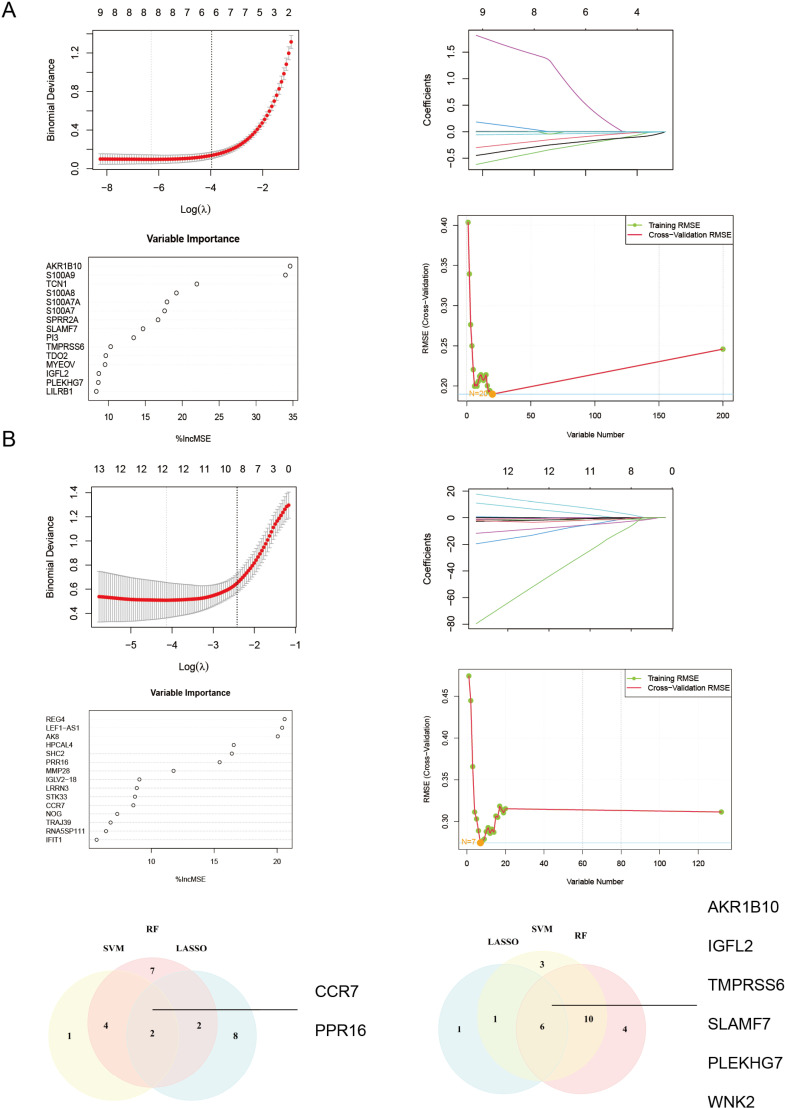
The screening process for hub genes of HS involved the LASSO model, the SVM-RFE model, the RF model, and their overlapping signature. A. Skin lesion; B. Peripheral blood.

To identify critical marker genes for HS, we employed the RF algorithm, LASSO logistic regression algorithm, and SVM-RFE algorithm. The results showed that 20 genes were identified with the RF algorithm, 8 genes determined by the LASSO logistic regression algorithm, and 20 genes were identified with the SVM-RFE algorithm (Fig 2A). Subsequent Venn diagrams highlighted that *AKR1B10, IGFL2, TMPRSS6, SLAMF7, PLEKHG7,* and *WNK2* were overlapping genes identified by all three algorithms. In the peripheral blood group, the LASSO regression algorithm screened 12 biomarkers, the SVM-RFE algorithm identified 7 biomarkers, and RF algorithm identified 15 biomarkers (Fig 2B). Ultimately, two genes—*PPR16* and *CCR7* were identified as key biomarkers across all three algorithms.

**Fig 3:**
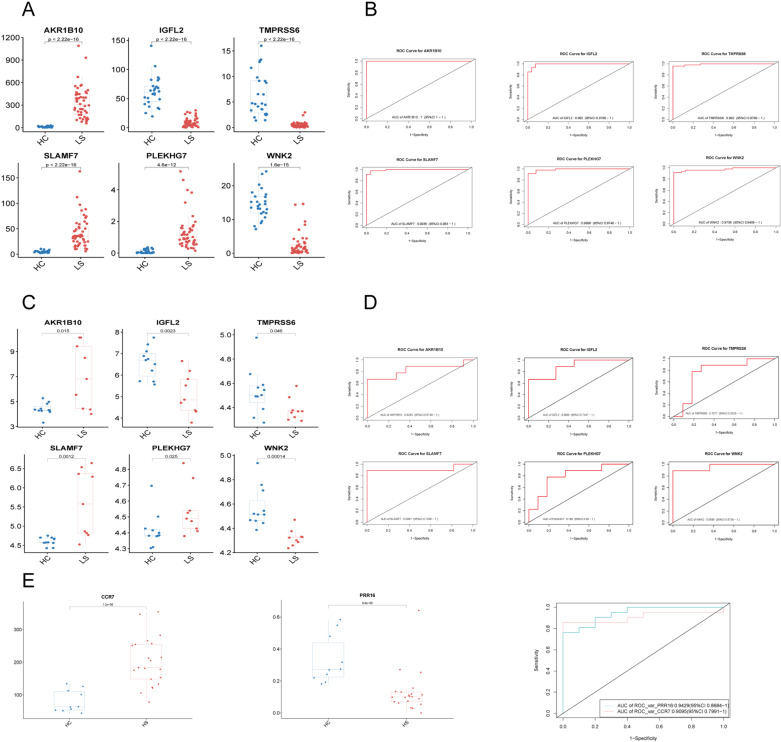
Results of diagnostic value assessment and gene expression. A. Expression of hub genes in HS patients compared to normal controls in skin lesion. B. The ROC curve of each candidate genes showed the HS diagnostic value. C&D. The ROC curve and expression of signatures in validation group. E. The expression and ROC curve of hub genes in Peripheral blood. ROC: Receiver Operating Characteristic.

Following the identification of these six genes, we conducted an initial analysis of their expression levels, revealing a significant difference in expression between HS and the healthy control group (Fig 3A). To validate these findings, we employed the GSE128637 dataset as a validation cohort to confirm the accuracy of the previous analytic results and assess the expression levels of the six biomarkers. In the validation cohort, *TMPRSS6* showed no significant difference in expression between HS lesions and healthy control skin (Fig 3C). However, IGFL2 and WNK2 were notably downregulated, while AKR1B10, SLAMF7, and PLEKHG7 were significantly upregulated in HS samples.

For a more comprehensive assessment of the diagnostic efficacy of *AKR1B10, IGFL2, SLAMF7, PLEKHG7*, and *WNK2*, we conducted validation using both the merged data and the GSE128637 dataset, employing Receiver Operating Characteristic (ROC) curves, considered the gold standard for evaluating diagnostic accuracy. The AUCs of the selected biomarkers were: *AKR1B10* (1.00, 95% CI: 1.00 − 1.00), IGFL2 (0.99, 95% CI: 0.98 − 1.00), *SLAMF7* (0.99, 95% CI:0.98 − 1), *PLEKHG7* (0.99, 95% CI: 0.98 − 1.00) and *WNK2* (0.97, 95% CI: 0.94 − 1.00) (Fig 3B). Furthermore, the AUCs of these genes were in the validation group, *AKR1B10* (0.82, 95% CI: 0.61–1.00), *IGFL2* (0.89, 95% CI: 0.74–1.00), *SLAMF7* (0.91, 95% CI: 0.73–1.00), *PLEKHG7* (0.80, 95% CI: 0.59–1.00) and *WNK2* (0.96, 95% CI: 0.87–1.00), Notably, these biomarkers demonstrated a high diagnostic value in both the HS training and validation groups, with AUCs predominantly greater than 0.8, indicating robust diagnostic potential (Fig 3C). In the peripheral blood group, the AUCs for the selected peripheral blood biomarkers were *PPR16* (0.943, 95% CI: 0.868–1) and *CCR7* (0.910, 95% CI: 0.799–1) (Fig 3D).

### Analysis of immune cell infiltration by CIBESORT Algorithm

There is growing clinical evidence and substantial experimental data supporting the significant role of immune mechanisms in the pathogenesis of HS [1,3,4]. To further explore these mechanisms, we performed a CIBERSORT algorithm to analyze the 22 immune cell phenotypes infiltration in HS and normal healthy people.

**Fig 4:**
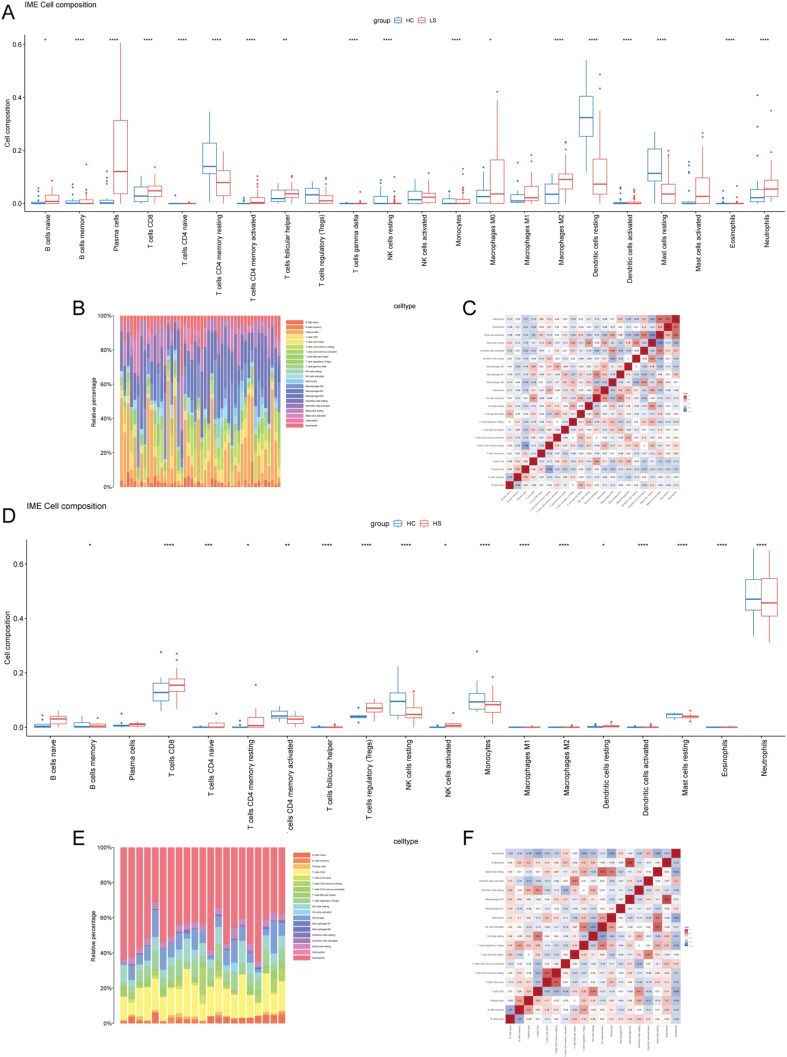
Distribution and visualization of immune cell infiltration. A&C. Comparison of 22 immune cell subtypes in HS and normal tissues. B&E. The distribution of 22 immune cell types in HS patients. C&F. Heatmap of correlations between 22 immune cell subtypes. A, B and C. Skin lesion; E, F and G. Peripheral blood.

We evaluated the heterogeneity of cell composition in both the HS and the healthy control samples. The findings revealed significant differences in immune cell infiltrates between the two groups (Fig 4A and 4D). The distinctive infiltration patterns of these immune cells suggest potential regulatory targets for treating HS.

Moreover, the bar charts visually depict the content of various subpopulations in each HS sample (Fig 4B and 4E). We then examined the correlations among the 22 immune cell types across all samples. Notably, there was a significant positive correlation between Neutrophils and Eosinophils (r = 0.67) and a significant negative correlation between T cells CD4 memory resting activated and Plasma cells (r = −0.56) in both HS lesion and normal samples (Fig 4B). In the peripheral blood group, NK cells resting exhibited a significant negative correlation with NK cells activated (r = -0.57), whereas Eosinophils showed a significant positive correlation with Macrophages M2 (r = 0.83) (Fig 4E). These correlations provide insights into the interplay among different immune cell types and may contribute to a better understanding of the immunological dynamics in HS.

### Expression of key signatures in single cells by scRNA‑seq analysis

**Fig 5:**
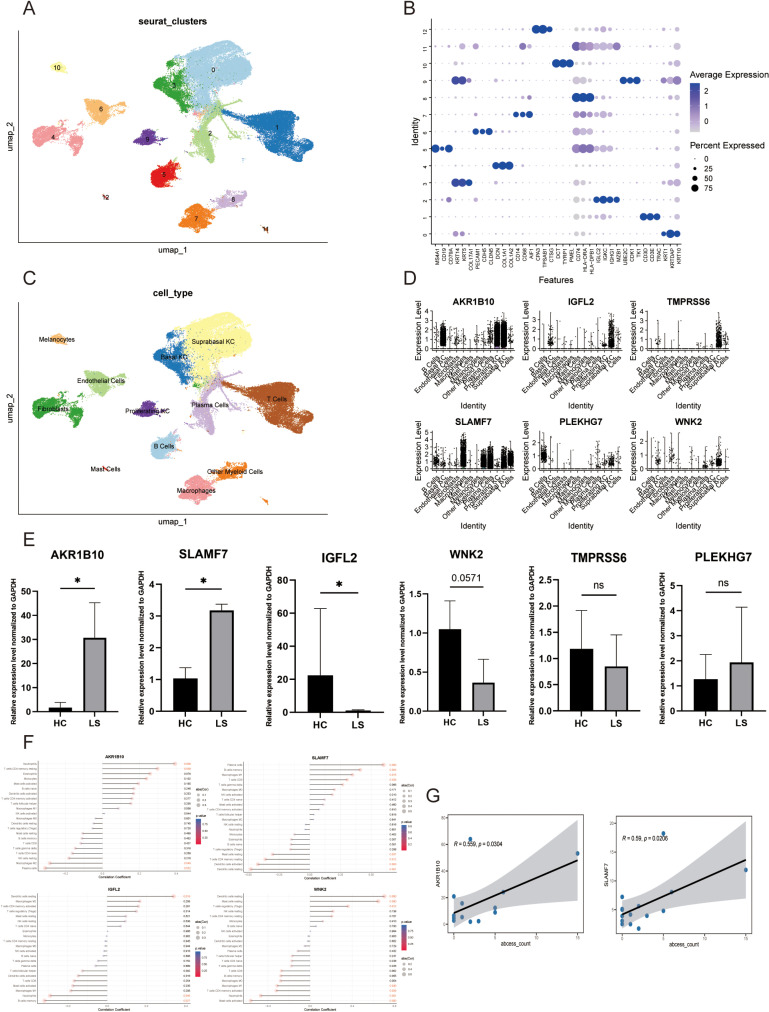
Hub genes expression analysis in the single-cell transcriptome, validation in our samples, and correlation analysis. A. Uniform Manifold Approximation and Projection (UMAP) plot of the 12 identified main cell types among single-cell transcriptome. B. Dot plot showing the expression of discriminatory markers for each cell type cluster. C. UMAP plot for 67,378 cells coloured by cell type cluster. D. Hub genes expression analysis in main cell types. E. Expression of hub genes in our study cohort(n = 6). F&G. The correlation of HS skin tissue key signatures with infiltrating immune cells and abscess count was analyzed. *: P < 0.05; ns: P > 0.05.

The resulting quality-controlled HS single-cell atlas comprised 67,378 cells, with an average of 2421 genes and 10,953 transcripts detected per cell. To investigate cellular heterogeneity, we selected variable genes and performed Uniform Manifold Approximation and Projection (UMAP) dimensionality reduction and cell clustering (Fig 5A). Cluster annotation was corroborated by overlapping the cluster marker genes with canonical cell type-defining signature genes. We identified 11 primary cell types including Basal KC (*KRT14, KRT5, COL17A1*), Suprabasal KC (*KRT1, KRTDAP, KRT10*), Proliferating KC (*UBE2C, CDK1, KRTK1*), Melanocytes (*DCT, TYRP1, PMEL*), B Cells (*MS4A1, CD19, CD79A*), Endothelial Cells (*PECAM1, CDH5, CLDN5*), Fibroblasts (*DCN, COL1A1, COL1A2*), T cells (*CD3D, CD3E, TRAC*), Mast Cells (*CPA3, TPSAB1, CTSG*), Macrophages(*CD14, CD68, AIF1*), Other Myeloid Cells(*CD74, HLA-DRA, HLA-DPB1*) and Plasma Cells (*IGLC2, IGKC, IGHG1, MZB1*) (Fig 5B). For a more precise characterization of the expression of key signatures within immune cells, we interrogated a scRNA-seq database to identify the cell populations expressing *AKR1B10, IGFL2, TMPRSS6, SLAMF7, PLEKHG7* and *WNK2* in HS lesions (Fig 5D).

### Expression of hub genes in HS patient

RT-PCR was performed on skin tissue samples obtained from HS surgeries and abdominoplasty procedures. This analysis confirmed the gene expression levels of these hub genes, *AKR1B10, IGFL2, TMPRSS6, SLAMF7, PLEKHG7* and *WNK2*. Consistent with the data analysis, our results demonstrated an upregulation of *AKR1B10* and *SLAMF7*, as well as a downregulation of *IGFL2* and *WNK2* expression in the HS lesions. However, *TMPRSS6* and *PLEKHG7* showed not significant changes in expression (Fig 5E).

### Correlation analysis between key signatures and infiltration‑related immune cells and abscess count

We conducted a Spearman correlation analysis to examine the relationship between key signatures and immune cell infiltration in HS patients (Fig 5F). In HS lesions, *AKR1B10* was positively correlated with Neutrophils (r = 0.39, p = 0.006), Resting CD4 memory T cells (r = 0.3, p = 0.039) and negatively correlated with Plasma cells (r = -0.31, p = 0.032), M2 Macrophages (r = -0.29, p = 0.049). *SLAMF7* was positively correlated with Plasma cells (r = 0.6, p ＜ 0.001), Memory B cells (r = 0.41, p = 0.004) M1 Macrophages (r = 0.35, p = 0.015), CD8 T cells (r = 0.3, p = 0.035), and negatively correlated with Resting Dendritic cells (r = -0.48, p = 0.001), Activated Dendritic cells (r = -0.42, p = 0.003), Resting CD4 memory T cells (r = -0.36, p = 0.012), Resting Mast cells (r = -0.3, p = 0.037). *WNK2* was positively correlated with Resting Dendritic cells (r = 0.71, p ＜ 0.001), Resting Mast cells (r = 0.65, p ＜ 0.001), Regulatory T cells (Tregs) (r = 0.36, p = 0.012), and negatively correlated with Activated Mast cells (r = -0.51, p ＜ 0.001), Neutrophils (r = -0.47, p = 0.001), Activated CD4 memory T cells (r = -0.32, p = 0.026), M1 Macrophages (r = -0.31, p = 0.030). *IGFL2* was positively correlated with Resting Dendritic cells (r = 0.34, p = 0.019), and negatively correlated with Memory B cells (r = -0.32, p = 0.027), Neutrophils (r = -0.29, p = 0.045). To further investigate the correlation between core genes and the disease progression, we conducted a Spearman correlation analysis to examine the relationship between hub genes and abscess count in HS patients (Fig 5G). *AKRB10* and *SLAMF7* were positively correlated with abscess count (r = 0.56, p = 0.03; r = 0.59, p = 0.02). *IGFL2* and *WNK2* showed negatively correlated with abscess count (r = -0.44, p = 0.1; r = -0.43, p = 0.11). These findings provide valuable insights into the complex interplay between the expression of hub genes and specific infiltrating immune cell populations in HS.

### The potential role of CCL19/CCL21-CCR7 axis in HS

**Fig 6:**
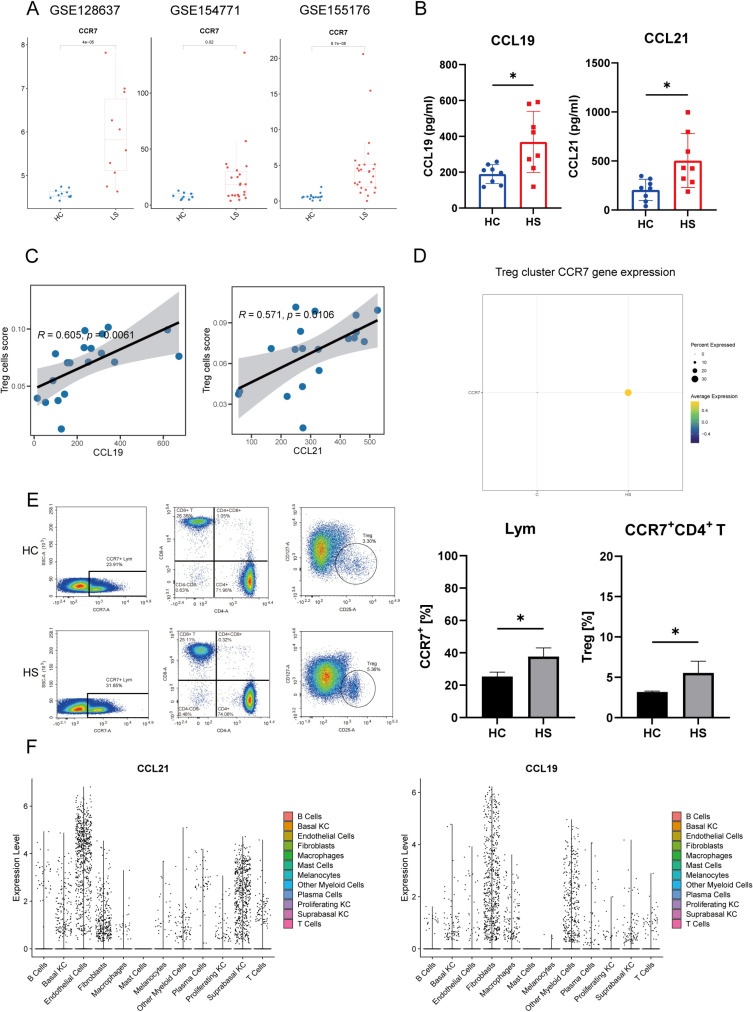
The potential role of CCL19 and CCL21 in hidradenitis suppurativa. A. CCR7 gene expression in HS lesion. B. Serum levels of CCL19 and CCL21 (n = 8). C. Correlation analysis between CCL19 and CCL21 in the skin and Treg cells in the blood. D. HS and healthy control average gene expression of Treg cluster. E. Flow cytometric analysis of CCR7 + lymphocytes percentages within lymphocytes compartment and Treg percentages within the CD4 + T cell compartment (n = 3). F. The CLL19 and CLL21 genes expression analysis in the HS cell subset clusters. (*P < 0.05, **P < 0.01, ***P < 0.005, ****P < 0.001).

In our study, *CCR7* was identified as a key gene in the blood and exhibits elevated expression in HS lesions, consistent with increased levels observed by flow cytometry (Fig 6A and 6E). *CCL19* and *CCL21* also showed higher expression in HS lesions compared to both non-lesional areas and skin from healthy [7]. Further analysis using ELISA confirmed that plasma levels of CCL19 and CCL21 were significantly elevated in HS patients (Fig 6B). Given the systemic nature of HS, we hypothesize that CCL19 and CCL21 secreted by various cells within skin lesions attracts circulating immune cells expressing CCR7 to the affected regions, thereby exacerbating skin inflammation. Notably, correlation analysis indicates a strong association between CCL19 and CCL21 in the lesions and Treg cells in the blood (Fig 6C). Through single-cell RNA sequencing and flow cytometry, we further validated our hypothesis, demonstrating that Treg cells in HS lesions express higher levels of CCR7 (Fig 6D and 6E). To elucidate the potential sources of CCL19 and CCL21 in the skin, single-cell sequencing revealed that *CCL19* is primarily expressed in fibroblasts and myeloid cells, while *CCL21* is predominantly enriched in non-immune cells such as keratinocytes and fibroblasts (Fig 6F). Based on these findings, we propose that the CCL19/CCL21-CCR7 axis plays a crucial role in the pathogenesis and progression of HS.

## Discussion

The pathogenesis of HS remains unclear, but there is a consensus that skin infiltrating immune cells play a pivotal role in its development. Most of the evidence has demonstrated a significant accumulation of various immune cells, including T cells, B cells, plasma cells, and macrophages in HS lesions [1,20,21]. Given the invasive nature of biopsies and the limited specificity of current treatment methods for HS, precise management remains a significant challenge, contributing to prolonged suffering among HS patients worldwide [22]. The imperative lies in the early identification of HS, timely intervention, and the improvement of HS patient’s quality of life [23]. There is an urgent need to develop novel biomarkers that can accurately predict the skin pathology of HS patients.

Epidemiological studies have underscored a significant familial aggregation of HS, suggesting a substantial influence of genetic factors on its development and progression. Nevertheless, a substantial portion of patients either lack identifiable genetic variations or specific mutated genes remain unidentified. While numerous genes have been implicated in HS pathogenesis [8–11], limited research has specifically addressed the aberrantly expressed gene associated with immune infiltration in comparisons between HS and healthy control blood and skin tissues. Therefore, our study identifies candidate hub genes for HS, investigate the role of immune cell infiltration, and demonstrates that CCL19 and CCL21, secreted by various cells within HS lesions, significantly attract circulating immune cells expressing CCR7, particularly Treg cells.

CCR7, also known as CD197, is a transmembrane protein with seven transmembrane domains. It facilitates the migration of immune cells, including B cells, naïve and central memory T cells, macrophage progenitors, NK cells, and mature dendritic cells (DCs), to secondary lymphoid organs by recognizing CCL19 and CCL21 [24,25]. In our study, we found that CCL19 and CCL21 are derived from fibroblasts and dendritic cells, recruiting CCR7-associated immune cells, particularly Treg cells. Previous studies have reported that tertiary lymphoid structures play a significant role in the pathogenesis of HS [26]. Interestingly, the CCL19/CCL21-CCR7 axis is involved in the pathogenesis of diseases such as psoriasis and multiple sclerosis, as well as in tertiary lymphoid structures [25]. We hypothesize that, in addition to CCR7-positive T regulatory cells, other CCR7-expressing immune cells may also be recruited from the bloodstream by CCL19 and CCL21 to participate in the pathogenesis of HS. CAP-100 (NCT04704323) and JBH492 (NCT04240704), monoclonal antibodies against CCR7, have been involved in Phase I clinical trials for Chronic lymphocytic leukemia and Non-Hodgkin lymphoma. Future research should further explore CCR7-targeted therapies in HS.

SLAMF7, alternatively referred to as CD319, belongs to the signaling lymphocytic activation molecule family. It is expressed on a diverse range of immune cells, including B cells, plasma cells, T cells, dendritic cells, NK cells, and monocytes, where it can mediate both activating and inhibitory functions [27]. However, SLAMF7 expression levels vary across immune cell types; it has high expression in plasma cells but exhibits low expression in inactive macrophages [28]. SLAMF7 is selectively expressed by macrophages from sites of inflammation and is regulated by IFN-γ in rheumatoid arthritis, inflammatory bowel disease, and COVID-19 pneumonia [29]. Interestingly, a study demonstrated that M1-polarized pro-inflammatory macrophages exhibit enhanced effector functions, with the STAT1/IFN-signaling axis and associated IFN-stimulated genes central to this dysregulation [30]. Consistent with these findings, our study demonstrated that SLAMF7 is prominently expressed in macrophages and is significantly associated with the M1 macrophage phenotype. Previous studies have demonstrated elevated IFN-γ and SLAMF7 expression in HS lesions compared to healthy controls [8,21]. These findings suggest that SLAMF7 regulation by IFN-γ in activated inflammatory macrophages represents a central pathway driving the HS pathology. Further functional studies using macrophage-specific SLAMF7 knockdown models may help elucidate its precise role in modulating inflammatory responses in HS.

AKR1B10 is a nicotinamide adenine dinucleotide phosphate (reduced coenzyme II)-dependent oxidoreductase with biological functions include carbonyl detoxification, hormone metabolism, osmotic adjustment, and lipid synthesis [31]. Several studies have reported a significant elevation of AKR1B family members in HS skin lesions [8,9,11]. Notably, AKR1B10 is a key enzyme involved in the expression of pro-inflammatory cytokines in SARS-CoV2 Severe Acute Respiratory Syndrome [32]. AKR1B10 has been shown to accelerate the production of proinflammatory cytokines in colon cancer [33]. Through single-cell transcriptomic analysis, we identified that AKR1B10 is predominantly expressed in keratinocytes and is significantly elevated in HS skin compared to healthy individuals. Given that keratinocytes act as vital pro-inflammatory mediators in HS, releasing substantial amounts of inflammatory cytokines during the early stages of the disease [34–36]. The specific mechanisms by which AKR1B10 contributes to keratinocyte-driven inflammation warrant further study, including its potential interactions with oxidative stress pathways and inflammasome activation.

IGFL2 is a member of the insulin-like growth factor family, essential signaling molecules that are pivotal for cellular energy metabolism, growth, and development, particularly during prenatal stages [37]. The study indicates that forty-nine percent of the patients exhibited significantly reduced IGF-1 levels, suggesting that HS patients with low IGF-1 levels may represent a distinct phenotype within the HS population [38]. Studies suggests that the expression levels of IGFL2 are diminished in psoriasis compared to healthy control skin [39]. Our research reveals that IGFL2 is primarily expressed in keratinocytes and exhibits a negative correlation with B cells and neutrophils, both of which play critical roles in HS pathogenesis [40]. Consequently, we propose that IGFL2 may modulate the inflammatory response via interactions between keratinocytes and immune cells.

WNK2, a cytoplasmic serine-threonine kinase within the protein kinase superfamily, plays pivotal roles in cell cycle progression, anti-apoptotic mechanisms, and metabolic regulation [41]. Studies suggest that WNK2-associated loci linked to susceptibility to inflammatory bowel disease also exhibit a significant association with pyoderma gangrenosum, both of which are comorbidities of HS [42,43]. In HS skin lesions, WNK2 exhibits predominantly expression in non-immune cells and demonstrates a negative correlation with several pro-inflammatory cells, including Activated Mast cells and Neutrophils. Concurrently, it shows a positive correlation with anti-inflammatory cells such as Resting Dendritic cells, Regulatory T cells, and Resting Mast cells. Thus, we postulate that WNK2 likely plays a pivotal role in modulating inflammation in HS. Exploring WNK2’s regulatory mechanisms in epidermal barrier function and immune tolerance may further clarify its protective role in HS pathogenesis.

Our study presents several innovative contributions. First, in our dataset selection, we incorporated both HS skin and blood datasets, enriching the breadth of our analysis. Notably, this dual dataset approach is unprecedented in HS research, setting our study apart from existing research focusing on singular datasets. Second, leveraging bioinformatics and three distinct machine learning techniques (LASSO, RF, and SVM-RFE), we identified *AKR1B10, IGFL2, WNK2, SLAMF7,* and *CCR7* as potential genes and therapeutic targets for HS. This integrative methodology enhances the robustness and reliability of our findings. Third, to corroborate our findings, we evaluated these genes in an independent GEO dataset, affirming the diagnostic efficacy of our gene-based model. This validation strengthens the credibility of our conclusions and provides a roadmap for future investigations into the molecular underpinnings of HS. Furthermore, our enrichment analysis and immune infiltration evaluation illuminate the nuanced relationship between HS, immunity, and inflammation. Notably, we found the potential role of the CCL19/CCL21-CCR7 axis in the pathogenesis of HS, targeting the CCL19/CCL21-CCR7 pathway may offer new therapeutic avenues for managing this debilitating condition. These findings introduce new perspectives for developing targeted therapies that go beyond conventional treatment paradigms, offering a promising foundation for precision medicine in HS. Collectively, these unique facets amplify the distinctiveness and depth of our research, laying a robust foundation for continued endeavors in comprehending and addressing HS.

## Conclusion

Our study utilized bioinformatics analysis of the GEO dataset to explore the underlying molecular mechanisms of HS and the immune cell infiltration landscape. Employing three machine learning algorithms (LASSO, RF, and SVM-RFE), we identified *AKR1B10, IGFL2, WNK2, SLAMF7,* and *CCR7* as potential biomarkers and therapeutic targets for HS treatment. Furthermore, our findings indicate that CCL19 and CCL21, secreted by various cells within HS lesions, significantly attract circulating immune cells expressing CCR7, particularly Treg cells. Targeting the CCL19/CCL21-CCR7 pathway may offer new therapeutic avenues for managing this debilitating condition. Future research should focus on further elucidating the molecular mechanisms underlying this axis and evaluating its potential as a biomarker for disease activity and therapeutic response.

## Supporting information

S1 Table Summary of patients. (PDF)
