# Supplementary material for: Bulk and single-cell RNA-sequencing analyses revealed potential key genes and the role of CCL19/CCL21-CCR7 axis in hidradenitis suppurativa
Source: PLoS One. 2025 Jun 2;20(6):e0322565. doi: 10.1371/journal.pone.0322565 (PMC12129208; doi:10.1371/journal.pone.0322565)
Supplement: S1 Table — (PDF) [file pone.0322565.s001.pdf]

Supplementary Table 1: Patient Characteristics

Patient	Gender	Hurley Stage	Sample
HS1	F	2	Plasma; Skin
HS2	F	2	Plasma; Skin
HS3	M	3	Plasma;PBMC; Skin
HS4	M	2	Plasma;PBMC; Skin
HS5	M	3	Plasma;PBMC; Skin
HS6	M	3	Plasma; Skin
HS7	M	2	Plasma
HS8	M	2	Plasma
HC1	F	NA	Plasma
HC2	M	NA	Plasma;PBMC
HC3	M	NA	Plasma
HC4	F	NA	Plasma
HC5	M	NA	Plasma;PBMC
HC6	M	NA	Plasma;PBMC
HC7	M	NA	Plasma
HC8	M	NA	Plasma
HC9	F	NA	Skin
HC10	M	NA	Skin
HC11	M	NA	Skin
HC12	F	NA	Skin
HC13	M	NA	Skin
HC14	M	NA	Skin
